# Preparative Separation of Spirobisnaphthalenes from Endophytic Fungus *Berkleasmium* sp. Dzf12 by High-Speed Counter-Current Chromatography

**DOI:** 10.3390/molecules181012896

**Published:** 2013-10-16

**Authors:** Tijiang Shan, Shiqiong Lu, Chao Luo, Ruiya Luo, Yan Mou, Mingan Wang, Youliang Peng, Ligang Zhou

**Affiliations:** 1 MOA Key Laboratory of Plant Pathology, Department of Plant Pathology, College of Agronomy and Biotechnology, China Agricultural University, Beijing 100193, China; 2 Department of Applied Chemistry, College of Science, China Agricultural University, Beijing 100193, China

**Keywords:** preparative separation, spirobisnaphthalenes, endophytic fungus, *Berkleasmium* sp. Dzf12, high-speed counter-current chromatogrphy

## Abstract

High-speed counter-current chromatography (HSCCC) was applied for the first time for the preparative separation of spirobisnaphthalenes from a crude extract of the endophytic fungus *Berkleasmium* sp. Dzf12, associated with the medicinal plant *Dioscorea zingiberensis*. Six spirobisnaphthalenes were successfully separated by HSCCC with a two-phase solvent system composed of *n*-hexane-chloroform-methanol-water (1.5:3.0:2.5:2.0, v/v). About 18.0 mg of diepoxin κ (**1**), 245.7 mg of palmarumycin C_13_ (**2**), 42.4 mg of palmarumycin C_16_ (**3**), 42.2 mg of palmarumycin C_15_ (**4**), 32.6 mg of diepoxin δ (**5**), and 22.3 mg of diepoxin γ (**6**) with purities of 56.82, 71.39, 76.57, 75.86, 91.01 and 82.48%, respectively, as determined by high-performance liquid chromatography (HPLC), were obtained from 500 mg of the crude extract in a one-step elution within 7 h of separation procedure by HSCCC. The purified spirobisnaphthalenes were further structurally characterized by means of physicochemical and spectrometric analysis.

## 1. Introduction

Spirobisnaphthalenes (also namely bisnaphthospiroketals) are a growing group of fungal metabolites which contain two 1,8-dihydroxynaphthalene-derived spiroketal units bridged through a spiroketal linkage [1,2]. Spirobisnaphthalenes possess a wide range of biological properties, including antibacterial [3,4,5], antifungal [6,7], algicidal [4,8], antiplasmodial [9], nematicidal [10], antileishmanial [11], cytotoxic [9] and anti-tumor [12] activities. Some of these compounds have been identified as novel inhibitors of rasfarnesyltransferase [13], DNA gyrase [14], topoisomerase II [15] and thioredoxin-reductase [16,17], and thus are of interest in terms of their potential in cancer chemotherapy.

In our previous study, a variety of bioactive spirobisnaphthalenes were isolated from the endophytic fungus *Berkleasmium* sp. Dzf12 derived from the medicinal plant *Dioscorea zingiberensis* [7,18]. This fungus was also found to be a high producer of spirobisnaphthalenes [19,20,21,22]. In order to speed up investigation and application of spirobisnaphthalenes in agriculture, medicine and food industry [23], one of the most important issues is how to efficiently obtain spirobisnaphthalenes from the fungal cultures.

High-speed counter-current chromatography (HSCCC), a support-free liquid-liquid partition chromatographic technique, eliminates the problem of irreversible adsorption of the sample on the solid support, and it offers the maximum capacity with an excellent sample recovery [24]. Moreover, HSCCC permits direct introduction of crude samples into the column without additional sample preparation. It has been successfully applied to the analysis and separation of various natural products such as flavonoids [24,25,26], alkaloids [27,28,29], polyphenols [30], terpenoids [31,32,33], and quinones [34] from medicinal plants and microorganisms.

To the best of our knowledge, there is no previous report on the preparative separation of spirobisnaphthalenes from fungi. The purpose of this study was to establish a method for more efficient separation and purification of spirobisnaphthalenes from the endophytic fungus *Berkleasmium* sp. Dzf12 cultures by HSCCC. The separated spirobisnaphthalenes were further purified by Sephadex LH-20 chromatography and preparative HPLC, and structurally characterized by means of physicochemical and spectrometric analysis.

## 2. Results and Discussion

### 2.1. HPLC Analysis of the Crude Extract

**Figure 1:**
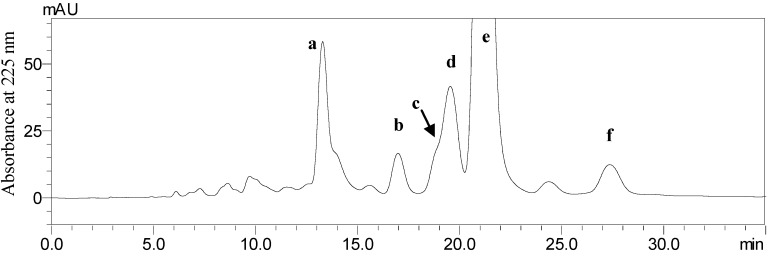
HPLC chromatogram of the ethyl acetate crude extract from endophytic fungus *Berkleasmium* sp. Dzf12. The retention times of peaks a–f were 13.28, 16.97, 18.54, 19.63, 21.13 and 27.34 min, respectively.

The crude ethyl acetate extract from the endophytic fungus *Berkleasmium* sp. Dzf12 was first analyzed by HPLC. The main spirobisnaphthalenes were satisfactorily separated with methanol-water (45:55, v/v) as the solvent system. The HPLC chromatogram of the ethyl acetate crude extract is shown in Figure 1. Peaks a, b, e and f correspond to palmarumycin C_15_ (**4**), diepoxin γ (**6**), palmarumycin C_13_ (**2**), and diepoxin κ (**1**), respectively. Peak c+d was a mixture of diepoxin δ (**5**) and palmarumycin C_16_ (**3**).

### 2.2. Selection of Two-Phase Solvent System for HSCCC

Successful separation by HSCCC largely depends upon the selection of a suitable two-phase solvent system, which was selected according to the partition coefficient (*K* value) of each target compound [35]. In general, an optimum *K* value should be within the range of 0.5–2.0 [36,37]. A smaller *K* value elutes the solute closer to the solvent front with lower resolution while a larger *K* value tends to give better resolution but broader, and more dilute peaks due to a longer elution time [35]. In this study, different solvent systems containing *n*-hexane-chloroform-methanol-water were examined to optimize the *K* values of the spirobisnaphthalenes by HPLC analysis. Their *K* values are shown in Table 1. The most appropriate *K* value was obtained at the volume ratio of 1.5:3.0:2.5:2.0 (v/v), which was selected to further isolate and purify the spirobisnaphthalenes by HSCCC in the present study.

**Table 1:** The partition coefficients (*K* values) of spirobisnaphthalenes in the two-phase solvent systems of *n*-hexane-chloroform-methanol-water by HPLC analysis.

No.	Ratio (v/v)	*K* value				
		Peak a	Peak b	Peak c+d	Peak e	Peak f
1	1.0:3.0:2.0:2.0	1.72	1.75	1.01	0.42	0.13
2	1.5:3.0:2.0:2.0	2.11	2.32	1.74	0.77	0.46
3	1.5:3.0:2.5:2.0	1.95	2.16	1.53	0.89	0.66
4	1.5:3.0:1.5:2.0	2.01	2.19	1.61	0.64	0.45
5	1.0:3.0:3.0:2.0	1.89	2.07	1.47	0.46	0.32

Note: “Ratio” is expressed as the volume ratio of *n*-hexane-chloroform-methanol-water. Peaks a–f in HPLC chromatogram correspond to compounds **4**, **6**, **5**, **3**, **2** and **1**, respectively.

### 2.3. Separation of Spirobisnaphthalenes by HSCCC and Structural Identification

**Figure 2:**
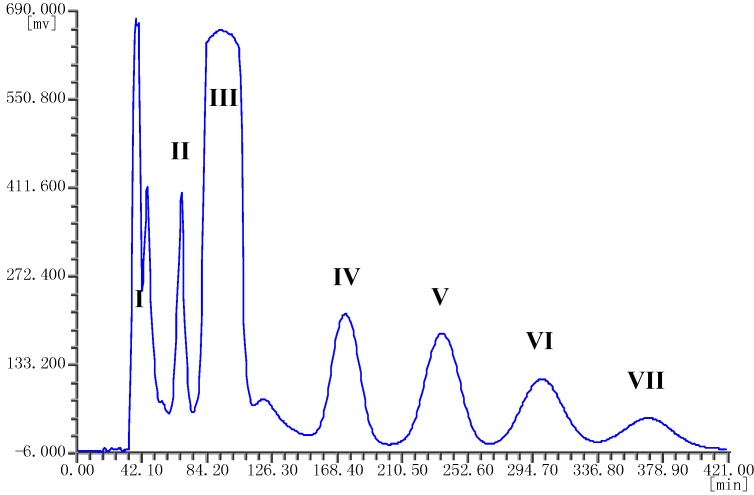
HSCCC chromatogram of the ethyl acetate crude extract from endophytic fungus *Berkleasmium* sp. Dzf12 with *n*-hexane-chloroform-methanol-water (1.5:3.0:2.5:2.0, v/v) solvent system.

The ethyl acetate extract of the endophytic fungus *Berkleasmium* sp. Dzf12 was fractionated by HSCCC using the optimized *n*-hexane-chloroform-methanol-water (1.5:3.0:2.5:2.0, v/v) solvent system. Seven peak fractions (*i.e*., I–VII) were obtained in one-step elution within 7 h. The HSCCC chromatogram is shown in Figure 2.

**Figure 3:**
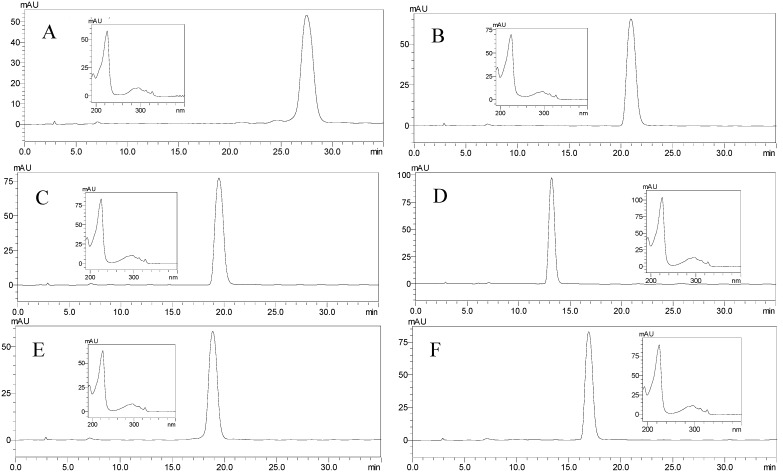
HPLC analysis and UV spectra of each HSCCC fraction. (**A**) diepoxin κ (**1**) from HSCCC peak fraction II; (**B**) palmarumycin C_13_ (**2**) from HSCCC peak fraction III; (**C**) palmarumycin C_16_ (**3**) from HSCCC peak fraction IV; (**D**) palmarumycin C_15_ (**4**) from HSCCC peak fraction V; (**E**) diepoxin δ (**5**) from HSCCC peak fraction VI; (**F**) diepoxin γ (**6**) from HSCCC peak fraction VII. The HPLC retention time of each HSCCC peak fraction (*i.e*., II–VII) was 27.48, 20.95, 19.15, 13.19, 18.87 and 16.92 min, respectively.

The separation was considered to be efficient with retention ratio (*S*_F_) of the stationary phase to be 73.68%. The HSCCC fractions were concentrated and further analyzed by HPLC which gave the chromatograms in Figure 3.

**Figure 4:**
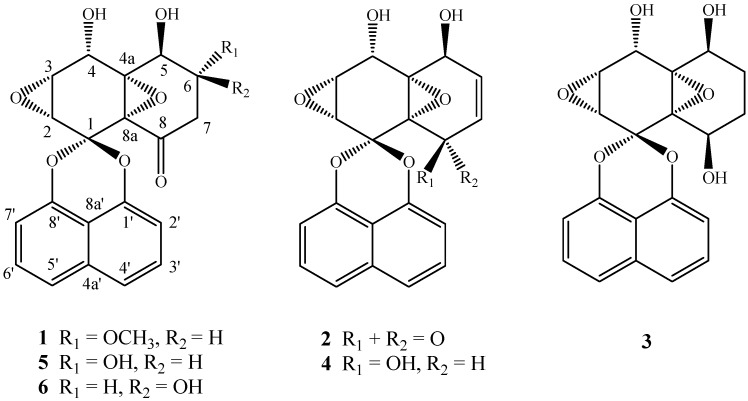
The structures of compounds **1**–**6**.

The HSCCC separation produced 18.0 mg of compound **1** with 56.82% purity in peak fraction II, 245.7 mg of compound **2** with 71.39% purity in peak fraction III, 42.4 mg of compound **3** with 76.57% purity in peak fraction IV, 42.2 mg of compound **4** with 75.86% purity in peak fraction V, 32.6 mg of compound **5** with 91.01% purity in peak fraction VI, 22.3 mg of compound **6** with 82.48% purity in peak fraction VII, respectively, from 500 mg of crude ethyl acetate extract only in one HSCCC run. In addition, concentrated peak fraction I was 46.9 mg, and the residue in the solenoid was 34.9 mg. So the sample recovery was 97.0%. After comparing their physicochemical and spectrometric data with those reported in the literatrure [4,38], the components were identified as diepoxin κ (**1**), palmarumycin C_13_ (**2**), palmarumycin C_16_ (**3**), palmarumycin C_15_ (**4**), diepoxin δ (**5**), and diepoxin γ (**6**), respectively, whose structures are shown in Figure 4. They are all belong to the deoxypreussomerin-type of spirobisnaphthalenes [2].

## 3. Experimental

### 3.1. General Analytical Methods

Preparative HSCCC was carried out with a model TBE-300B instrument (Tauto Biotech, Shanghai, China). The apparatus was equipped with a polytetrafluoroethylene tube (diameter of tube was 2.6 mm, and total volume was 280 mL) composed of three preparative coils and a 20-mL sample loop. The HSCCC system was equipped with a TBP-5002 pump and TBD-2000 UV detector operating at 280 nm (Tauto Biotech), and a WH500-USB workstation (Wuhao, Shanghai, China). The experimental temperature was 25 °C adjusted by HX-1050 constant temperature circulating implement (Boyikang, Beijing, China).

The analytical HPLC system (Shimadzu, Kyoto, Japan) consisted of two LC-20AT solvent delivery units, an SIL-20A autosampler, an SPD-M20A photodiode array detector, and CBM-20Alite system controller. Chromatographic separations were performed at 30 °C using Synergi reversed-phase Hydro-C_18_ column (250 mm × 4.6 mm, 5 μm, Phenomenex, Torrance, CA, USA). The mobile phase composed of methanol-water (45:55, v/v) was eluted at a flow rate of 1.0 mL/min, and the effluent was monitored at 225 nm. The LC solution multi-PDA workstation was employed to acquire and process chromatographic data. Total time of analysis was 35 min.

The preparative HPLC system consisted of K-501 pump, K-2501 UV detector (Knauer, Berlin, Germany), a 2 mL sample loop, a workstation (Lumtech, Beijing, China), and an Ultimate XB reversed-phase C_18_ column (21.2 mm × 250 mm, 5 μm, Welch Materials, Inc., Maryland, MD, USA).

Sephadex LH-20 was purchased from Pharmacia Biotech, Uppsala, Sweden. HR-ESI-MS spectra were measured on Bruker Apex IV FTMS. NMR spectra (^1^H-NMR and ^13^C-NMR) were recorded on a Bruker Avance DRX-400 NMR spectrometer (^1^H at 400 MHz and ^13^C at 100 MHz). The chemical shifts were expressed in ppm as *δ* values relative to tetramethylsilane (TMS) as an internal standard. Thin layer chromatography (TLC) plates were coated with 0.5-mm layers of silica gel (GF_254_, 300–400 mesh, Qingdao Marine Chemical Company, Qingdao, China). The melting point was determined on an XT4-100B microscopic melting-point apparatus (Tianjin Tianguang Optical Instruments Company, Tianjin, China).

### 3.2. Chemicals and Reagents

All organic solvents used for sample preparation and HSCCC were of analytical grade and purchased from Beijing Chemical Company (Beijing, China). Methanol used for preparative and analytical HPLC was chromatography grade and was purchased from Xilong Chemical Company (Guangdong, China). Water used was distilled. Other chemicals were of analytical reagent grade.

### 3.3. Preparaton of Crude Sample

The endophytic fungus Dzf12 was isolated from the medicinal plant *Dioscorea zingiberensis* and was identified as *Berkleasmium* sp. Dzf12 (accession number DQ280463 in the GenBank) through its morphological characteristics and internal transcribed spacer (ITS) rRNA gene sequence analysis [7]. It was stored both on PDA slants at 4 °C and in 40% glycerol at −70 °C in the Herbarium of the College of Agronomy and Biotechnology, China Agricultural University (Beijing, China). The fungus was cultured on PDA (potato 200 g/L, dextrose 20 g/L and agar 20 g/L) medium in Petri dishes at 25 °C for 10 days. For seed culture, two to three plugs of agar medium (0.5 cm × 0.5 cm) with fungal cultures were inoculated in each 250 mL Erlenmeyer flask containing 100 mL potato dextrose broth (PDB) medium, and incubated on a rotary shaker at 150 rpm and 25 °C for 5 days. For fermentation culture, about 50 mycelia pellets were inoculated in each 1 L Erlenmeyer flask containing 300 mL PDB medium, and incubated on a rotary shaker at 150 rpm and 25 °C for 15 days. Afterwards, a total of 10 L fermentation broth was harvested.

The mycelia were separated from the culture filtrate by filtration and dried and powdered, and then extracted for three times with methanol. The dried methanol extract was suspension with water and extracted with ethyl acetate for three times. The culture filtrate was concentrated and also extracted three times with ethyl acetate. All the ethyl acetate extractions were concentrated under vacuum at 40 °C on a rotary evaporator to obtain the crude extract, and a total of 8.0 g crude extract was obtained.

### 3.4. Solvent Systems for HSCCC

Two phase solvent systems containing different ratios of *n*-hexane, chloroform, methanol and water were prepared (Table 1). The partition coefficients (*K* values) were determined by HPLC and calculated according to the ratio of the compound concentration, *K* = *C*_stationaryphase_/*C*_mobilephase_, where *C*_stationary phase_ is the compound concentration in the stationary phase (upper phase), and *C*_mobilephase_ is the compound concentration in the mobile phase (lower phase). As the volumes of the upper and lower phases of the pre-equilibrated two-phase solvent system are equal, the K values are then determined according the ratio of the peak area, *K* = *A*_stationaryphase_/*A*_mobilephase_, where *A*_stationaryphase_ is the peak area of the target compound in the stationary phase (upper phase), and *A*_mobilephase_ is the peak area of the target compound in the mobile phase (lower phase). The HPLC analysis was performed using a reversed-phase C_18_ (250 mm × 4.6 mm, 5 μm) at 30 °C. The elution system consisted of solvent A (MeOH) and solvent B (H_2_O) at the volume ratio of 45:55 (v/v), elution was done at 1.0 mL/min. UV detection was at 225 nm.

### 3.5. HSCCC Separation Procedure

The *n*-hexane-chloroform-methanol-water (1.5:3.0:2.5:2.0, v/v) solvent system was equilibrated at room temperature in a separatory funnel and the two phases were separated shortly before use. The coil column was first entirely filled with the stationary phase (the upper phase) of the solvent system. Then the apparatus was rotated at 850 rpm, while the mobile phase (the lower phase) was pumped into the column at a flow rate of 3 mL/min. After the mobile phase front emerged, and hydrodynamic equilibrium was established in the column, about 20 mL of sample solution containing 500 mg of the ethyl acetate extract was injected through the injection valve. The effluent from the column outlet was monitored with a UV detector at 280 nm and fractions were collected manually according to the absorbance plot from the detector. The temperature of the apparatus was set at 25 °C.

The retention ratio of stationary phase was also calculated, and the equation was as follows:

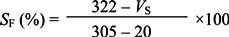
(1)
where *S*_F_ is the retention ratio of stationary phase (upper phase), and *V*_S_ is the volume of stationary phase flowing out. The yields were 46.9 mg (yield 9.38% of the ethyl acetate extract) for peak fraction I, 18.0 mg (yield 3.60%) for peak fraction II, 245.7 mg (yield 49.13%) for peak fraction III, 42.4 mg (yield 8.48%) for peak fraction IV, 42.2 mg (yield 8.44%) for peak fraction V, 32.6 mg (yield 6.53%) for peak fraction VI, and 22.3 mg (yield 4.46%) for peak fraction VII, respectively. Fraction I was examined by thin layer chromatography (TLC) to be complicated with many minor compounds.

### 3.6. Analysis and Identificaton of HSCCC Peak Fractions

Each peak fraction separated by HSCCC was further purified by Sephadex LH-20 chromatography and preparative HPLC. The ethyl acetate crude extract and peak fractions separated by HSCCC were analyzed by HPLC. The linear equations of the compounds by HPLC analysis were as follows: *Y* = 9175440.8889*X* − 161194.4444 (*R*^2^ = 0.9993) (for diepoxin κ, **1**), *Y* = 7914352.2614*X* + 17902.7516 (*R*^2^ = 0.9998) (for palmarumycin C_13_, **2**), *Y* = 9507067.6567*X* − 241729.0650 (*R*^2^ = 0.9986) (for palmarumycin C_16_, **3**), *Y* = 7659383.6667*X* – 71827.9417 (*R*^2^ = 0.9973) (for palmarumycin C_15_, **4**), *Y* = 6701365.6667*X* − 59984.1917 (*R*^2^ = 0.9997) (for diepoxin δ, **5**), *Y* = 7981014.8333*X* + 108748.6042 (*R*^2^ = 0.9986) (for diepoxin γ, **6**), where *Y* was the peak area, *X* was quality (μg) of the sample injected for each time, and *R* was the correlation coefficient. The physicochemical and spectrometric data of six spirobisnaphthalenes were given as follows.

*Diepoxin κ* (**1**). White needle-like crystals (MeOH); m.p. 230–232 °C; UV (MeOH) λ_max_ 225, 297, 312, and 327 nm. The molecular formula C_21_H_18_O_8_ was determined by HR-ESI-MS *m*/*z* 416.1339 ([M + NH_4_]^+^, calcd for C_21_H_22_O_8_N, 416.1339). ^1^H-NMR (DMSO-*d*_6_) *δ* (ppm): 7.63 (1H, d, *J*_3′,4′_ = 2.2 Hz, H-4′), 7.61 (1H, d, *J*_5′,6′_ = 2.2 Hz, H-5′), 7.53 [1H, pseudo-t (dd), *J*_2′,3′_ = 7.7 Hz, *J*_3′,4′_ = 8.2 Hz, H-3′], 7.50 [1H, pseudo-t (dd), *J*_5′,6′_ = 7.8 Hz, *J*_6′,7′_ = 8.2 Hz, H-6′], 7.08 (1H, d, *J*_2′,3′_ = 7.4 Hz, H-2′), 7.04 (1H, d, *J*_6′,7′_ = 7.4 Hz, H-7′), 6.07 (1H, d, *J*_5,5-OH_ = 5.8 Hz, OH-5), 5.91 (1H, d, *J*_4,4-OH_ = 7.6 Hz, OH-4), 4.76 (1H, d, *J*_3,4_ = 5.8 Hz, H-4), 4.35 (1H, dd, *J*_5,6_ = 4.0 Hz, H-5), 3.52–3.55 (1H, m, H-6), 3.35 (2H, d, *J* = 7.7 Hz, H-2, H-3), 3.27 (3H, s, OCH_3_-9), 2.60-2.62 (2H, m, H-7); ^13^C-NMR (DMSO-*d*_6_) *δ* (ppm): 95.3 (C-1), 52.6 (C-2), 55.2 (C-3), 60.9 (C-4), 70.1 (C-4a), 63.9 (C-5), 79.0 (C-6), 40.2 (C-7), 198.3 (C-8), 62.7 (C-8a), 56.6 (OCH_3_-9), 145.2 (C-l′), 109.1 (C-2′), 127.8 (C-3′), 120.7 (C-4′), 133.7 (C-4a′), 120.7 (C-5′), 127.7 (C-6′), 108.8 (C-7′), 145.5 (C-8′), 111.6 (C-8a′). The structure was confirmed by comparison with the literature data [38].

*Palmarumycin C_13_* (**2**). White needle-like crystals (MeOH); m.p. 162–164 °C; UV (MeOH) λ_max_ 225, 297, 312, and 327 nm. The molecular formula C_20_H_14_O_7_ was determined by HR-ESI-MS *m*/*z* 405.0372 ([M + K]^+^, calcd for C_20_H_14_O_7_K, 405.0372). ^1^H-NMR (DMSO-*d*_6_) *δ* (ppm): 7.64 (lH, d, *J* = 7.4 Hz, H-4′), 7.62 (lH, d, *J* = 7.4 Hz, H-5′), 7.49−7.60 [2H, pseudo-t (dd), H-3′, H-6′], 7.10 (1H, d, *J* = 7.5 Hz, H-2′), 7.04 (1H, d, *J* = 7.6 Hz, H-7′), 6.74 (1H, dd, *J* = 4.9, 10.5 Hz, H-6), 6.24 (1H, d, *J* = 7.8 Hz, OH-4), 6.00 (1H, d, *J* = 7.5 Hz, OH-5), 5.88 (1H, d, *J* = 2.2, 10.5 Hz, H-7), 4.98 (lH, d, *J* = 7.6 Hz, H-4), 4.67-4.70 (1H, m, H-5), 3.17 (2H, d, *J* = 5.2 Hz, H-2, H-3); ^13^C-NMR (DMSO-*d*_6_) *δ* (ppm): 95.1 (C-1), 52.7 (C-2), 55.2 (C-3), 59.7 (C-4), 70.7 (C-4a), 60.6 (C-5), 125.3 (C-6), 144.8 (C-7), 188.8 (C-8), 62.2 (C-8a), 145.2 (C-l′), 109.1 (C-2′), 127.9 (C-3′), 120.7 (C-4′), 133.8 (C-4a′), 120.7 (C-5′), 127.7 (C-6′), 108.7 (C-7′), 145.5 (C-8′), 111.5 (C-8a′). The structure was confirmed by comparison with the literature data [4,38].

*Palmarumycin C_16_* (**3**). Colorless waxy solid (MeOH); m.p. 187–188 °C; UV (MeOH) λ_max_ 225, 297, 312, and 327 nm. The molecular formula C_20_H_18_O_7_ was determined by HR-ESI-MS *m*/*z* 393.0944 ([M + Na]^+^, calcd for C_20_H_18_O_7_Na, 393.0945). ^1^H-NMR (CD_3_OD) *δ* (ppm): 7.58 (1H, d, *J*_3′,4′_ = 8.2 Hz, H-4′), 7.55 (1H, d, *J*_5′,6′_ = 8.2 Hz, H-5′), 7.44−7.51 (2H, m, H-3′, H-6′), 7.11 (1H, d, *J*_2′,3′_ = 7.4 Hz, H-2′), 7.02 (1H, d, *J*_6′,7′_ = 7.4 Hz, H-7′), 4.77 (1H, d, *J*_3,4_ = 2.7 Hz, H-4), 4.65 (1H, m, H-8), 4.44 (1H, m, H-5), 3.42 (1H, d, *J*_2,3_ = 4.2 Hz, H-2), 3.38 (1H, dd, *J*_2,3_ = 4.3 Hz, *J*_3,4_ = 2.8 Hz, H-3), 1.91 (2H, m, H-6eq, H-7eq), 1.45 (2H, m, H-6ax, H-7ax); ^13^C-NMR (CD_3_OD) *δ* (ppm): 98.1 (C-1), 53.7 (C-2), 56.9 (C-3), 62.9 (C-4), 69.0 (C-4a), 64.0 (C-5), 23.7 (C-6), 25.7 (C-7), 63.0 (C-8), 67.7 (C-8a), 147.4 (C-l′), 111.1 (C-2′), 128.8 (C-3′), 122.2(C-4′), 135.6 (C-4a′), 122.2 (C-5′), 128.6 (C-6′), 110.2 (C-7′), 147.7 (C-8′), 114.0 (C-8a′). The structure was confirmed by comparison with the literature data [4].

*Palmarumycin C_15_* (**4**). Colorless waxy solid (MeOH); m.p. 150–152 °C; UV (MeOH) λ_max_ 225, 297, 312, and 327 nm. The molecular formula C_20_H_16_O_7_ was determined by HR-ESI-MS *m*/*z* 391.0784 ([M + Na]^+^, calcd for C_20_H_16_O_7_Na, 391.0789). ^1^H-NMR (CD_3_OD) *δ* (ppm): 7.55 (2H, d, *J* = 8.4 Hz, H-4′, H-5′), 7.44-7.50 (2H, m, H-3′, H-6′), 7.09 (1H, d, *J*_2′,3′_ = 7.4 Hz, H-2′), 7.01 (1H, d, *J*_6′,7′_ = 7.5 Hz, H-7′), 5.75 (1H, dd, *J*_6,7_ = 10.4 Hz, *J*_5,6_ = 4.5 Hz, *J*_6,8_ = 1.7 Hz, H-6), 5.57 (1H, dd, *J*_6,7_ = 10.5 Hz, *J*_7,8_ = 2.6 Hz, H-7), 4.96 (1H, s, H-8), 4.93 (1H, d, *J*_3,4_ = 2.8 Hz, H-4), 4.53 (1H, d, *J*_4,5_ = 4.5 Hz, H-5), 3.47 (1H, d, *J*_2,3_ = 4.3 Hz, H-2), 3.42 (1H, dd, *J*_2,3_ = 4.3 Hz, *J*_3,4_ = 2.8 Hz, H-3); ^13^C-NMR (CD_3_OD) *δ* (ppm): 97.9 (C-1), 54.4 (C-2), 57.1 (C-3), 62.6 (C-4), 68.9 (C-4a), 62.5 (C-5), 126.7 (C-6), 129.4 (C-7), 64.3 (C-8), 67.4 (C-8a), 147.3 (C-l′), 111.0 (C-2′), 128.9 (C-3′), 122.2(C-4′), 135.6 (C-4a′), 122.0 (C-5′), 128.6 (C-6′), 110.2 (C-7′), 147.7 (C-8′), 114.2 (C-8a′). The structure was confirmed by comparison with the literature data [4].

*Diepoxin δ* (**5**). White needle-like crystals (MeOH); m.p. 241 °C; UV (MeOH) λ_max_ 225, 297, 312, and 327 nm. The molecular formula C_20_H_16_O_8_ was determined by HR-ESI-MS *m*/*z* 402.1180 ([M + NH_4_]^+^, calcd for C_20_H_20_O_8_N, 402.1183), and HR-ESI-MS *m*/*z* 423.0473 ([M + K]^+^, calcd for C_20_H_16_O_8_K, 423.0744). ^1^H-NMR (DMSO-*d*_6_) *δ* (ppm): 7.62 (1H, d, *J*_3′,4′_ = 8.0 Hz, H-4′), 7.60 (1H, d, *J*_5′,6′_ = 8.0 Hz, H-5′), 7.48-7.55 [2H, pseudo-t (dd), H-3′, H-6′), 7.09 (1H, d, *J*_2′,3ʹ_ = 7.5 Hz, H-2′), 7.05 (1H, d, *J*_6′,7′_ = 7.5 Hz, H-7′), 5.94 (1H, d, *J*_5,5-OH_ = 5.7 Hz, OH-5), 5.86 (1H, d, *J*_4,4-OH_ = 7.4 Hz, OH-4), 5.04 (1H, d, *J*_6,6-OH_ = 5.6 Hz, OH-6), 4.78 (1H, d, *J*_3,4_ = 7.5 Hz, H-4), 4.17-4.19 (1H, m, H-5), 3.77–3.83 (1H, m, H-6), 3.41 (2H, m, H-2, H-3), 2.53 (2H, d, *J*_6,7_ = 5.6 Hz, H-7); ^13^C-NMR (DMSO-*d*_6_) *δ* (ppm): 95.3 (C-1), 52.6 (C-2), 55.3 (C-3), 61.0 (C-4), 70.4 (C-4a), 66.8 (C-5), 69.6 (C-6), 42.9 (C-7), 198.8 (C-8), 62.9 (C-8a), 145.3 (C-l′), 109.1 (C-2′), 127.9 (C-3′), 120.8(C-4′), 133.8 (C-4a′), 120.7 (C-5′), 127.7 (C-6′), 108.8 (C-7′), 145.5 (C-8′), 111.6 (C-8a′). The structure was confirmed by comparison with the literature data [38].

*Diepoxin γ* (**6**). White needle-like crystals (MeOH); m.p. 176-178 °C; UV (MeOH) λ_max_ 225, 297, 312, and 327 nm. The molecular formula C_20_H_16_O_8_ was determined by HR-ESI-MS *m*/*z* 404.0738 ([M + Na]^+^, calcd for C_20_H_16_O_8_Na, 404.0737). ^1^H-NMR (acetone-*d*_6_) *δ* (ppm): 7.59 (1H, d, *J*_3′,4′_ = 2.3 Hz, H-4′), 7.57 (1H, d, *J*_5′,6′_ = 2.3 Hz, H-5′), 7.52 [1H, pseudo-t (dd), *J*_5′,6′_ = 7.8 Hz, *J*_6′,7′_ = 8.0 Hz, H-3′], 7.49 [1H, pseudo-t (dd), *J*_5′,6′_ = 7.8 Hz, *J*_6′,7′_ = 8.0 Hz, H-6′], 7.02 (1H, d, *J*_2′,3′_ = 7.5 Hz, H-2′), 6.97 (1H, d, *J*_6′,7′_ = 7.6 Hz, H-7′), 4.97 (1H, d, *J*_5,5-OH_ = 7.0, OH-5), 4.90 (2H, br. s, OH-4, OH-6), 4.72 (1H, d, H-4), 4.22 (1H, d, H-5), 4.10 (1H, m, H-6), 3.47–3.48 (1H, m, H-3), 3.43 (1H, d, *J*_2,3_ = 4.1, H-2), 2.53-2.55 (2H, m, H-7); ^13^C-NMR (acetone-*d*_6_) *δ* (ppm): 96.3 (C-1), 54.2 (C-2), 56.1 (C-3), 63.0 (C-4), 70.5 (C-4a), 67.2 (C-5), 63.2(C-6), 42.6 (C-7), 197.3 (C-8), 64.8 (C-8a), 146.7 (C-l′), 109.8 (C-2′), 128.6 (C-3′), 121.5 (C-4′), 135.2 (C-4a′), 121.5 (C-5′), 128.4 (C-6′), 109.5 (C-7′), 146.9 (C-8′), 113.0 (C-8a′). The structure was confirmed by comparison with literature data [38].

## 4. Conclusions

The preparative separation of spirobisnaphthalenes from the endophytic fungus *Berkleasmium* sp. Dzf12 by classical methods is tedious, time consuming, and requires multiple chromatographic steps on silica gel, Sephadex LH-20, C_18_ reversed-phase silica gel, *etc.* [7]. In this work, six spirobisnaphthalenes, including diepoxin κ (**1**), palmarumycin C_13_ (**2**), palmarumycin C_16_ (**3**), palmarumycin C_15_ (**4**), diepoxin δ (**5**), and diepoxin γ (**6**), were successfully separated in one step from an endophytic fungus *Berkleasmium* sp. Dzf12 by HSCCC with a *n*-hexane-chloroform-methanol-water (1.5:3.0:2.5:2.0, v/v) two-phase solvent system. This is the first report on the application of HSCCC for the separation of spirobisnaphthalenes from the cultures of endophytic fungus *Berkleasmium* sp. Dzf12. If an HPLC method was used for preparative separation of spirobisnaphthalenes, diepoxin δ (**5**) and palmarumycin C_16_ (**3**) could not be separated (Figure 1). Moreover, the amounts of the spirobisnaphtalenes obtained by preparative HPLC were less than by HSCCC. The present study will provide a basis for a large preparation of spirobisnaphthalenes from endophytic fungus *Berkleasmium* sp. Dzf12, and also demonstrates that HSCCC is an efficient technique in preparatively separating bioactive compounds from fungi. More research work should be devoted to screening an alternative two-phase solvent system of HSCCC in order to shorten the time required to isolate the spirobisnaphthalenes.
